# Spectroscopic Studies of Dual Fluorescence in 2-(4-Fluorophenylamino)-5-(2,4-dihydroxybenzeno)-1,3,4-thiadiazole: Effect of Molecular Aggregation in a Micellar System

**DOI:** 10.3390/molecules23112861

**Published:** 2018-11-02

**Authors:** Grzegorz Czernel, Arkadiusz Matwijczuk, Dariusz Karcz, Andrzej Górecki, Agnieszka Niemczynowicz, Aleksandra Szcześ, Grzegorz Gładyszewski, Alicja Matwijczuk, Bożena Gładyszewska, Andrzej Niewiadomy

**Affiliations:** 1 Department of Biophysics, University of Life Sciences in Lublin, Akademicka 13, 20-950 Lublin, Poland; grzegorz.czernel@up.lublin.pl (G.C.); alicjakruk@vp.pl (A.M.); bozena.gladyszewska@up.lublin.pl (B.G.); 2 Department of Analytical Chemistry (C1), Faculty of Chemical Engineering and Technology, Krakow Technical University, Warszawska 24, 31-155 Krakow, Poland; dariuszkarcz@indy.chemia.pk.edu.pl; 3 Department of Physical Biochemistry, Faculty of Biochemistry, Biophysics and Biotechnology of the Jagiellonian University, Gronostajowa 7, 30-387 Krakow, Poland; andrzej.gorecki@uj.edu.pl; 4 Department of Analysis and Differential Equations, Faculty of Mathematics and Computer Science, University of Warmia and Mazury, Słoneczna 54, PL-10-710 Olsztyn, Poland; aga.niemczynowicz@gmail.com; 5 Department of Physical Chemistry–Interfacial Phenomena, Faculty of Chemistry, Maria Curie–Sklodowska University, 20-031 Lublin, Poland; aszczes@poczta.umcs.lublin.pl; 6 Department of Applied Physics, Lublin University of Technology, Nadbystrzycka 38, 20-618 Lublin, Poland; g.gladyszewski@pollub.pl; 7 Institute of Industrial Organic Chemistry, Annopol 6, 03-236 Warsaw, Poland; andrzej.niewiadomy@up.lublin.pl; 8 Department of Chemistry, University of Life Sciences in Lublin, 20-950 Lublin, Poland

**Keywords:** 1,3,4-thiadiazole, molecular spectroscopy, molecular aggregation, induced dual fluorescence effects, micellar systems

## Abstract

The article presents the results of spectroscopic studies focused on a selected compound from the 1,3,4-thiadiazole group—2-(4-fluorophenylamino)-5-(2,4-dihydroxybenzeno)-1,3,4-thia-diazole (FABT)—in a micellar system formed by Triton X-100, a non-ionic detergent. Fluorescence measurements revealed the phenomenon of dual fluorescence whose emergence is related to the particular molecular organisation of the compound, which depends both on the concentration of the detergent and, most of all, the concentration of the compound itself. Dual fluorescence of FABT in a micellar system was observed for the compound dissolved in a methanol aqueous system, i.e., an environment wherein the dual fluorescence of the compound had never been reported before. Based on the interpretation of UV-Vis electronic absorption, resonance light scattering (RLS), emission and excitation fluorescence spectra, as well as measurements of dynamic light scattering (DLS) and Principal Component Analysis (PCA), we were able to relate the occurrence of this effect to the process of molecular aggregation taking place between FABT molecules in the micellar system in question. Results of fluorescence spectra measurements and time-correlated single photon counting (TCSPC) indicate that dual fluorescence occurs at detergent concentrations necessary to form micellar systems, which in turn facilitate the process of aggregation of FABT molecules. The correlation between the observed fluorescence effects and the previous measurements performed for analogues from this group suggests the possibility of charge transfer (CT) within the range of detergent concentrations wherein the aforementioned fluorescence effects are observed. It ought to be emphasised that this type of fluorescence effects are relatively easy to induce, which predisposes this groups of fluorophores as ideal fluorescence probes in the context of biological samples.

## 1. Introduction

Among the currently researched molecules, a group of particular interest to photochemists and physicists includes particles with photophysical properties related to the phenomenon of dual fluorescence. The mechanisms responsible for the spectroscopic properties of such molecules tend to contradict certain well established theories, e.g., Kasha’s principle [1,2,3]. The phenomenon’s significance is evident particularly in the physicochemical and spectroscopic perspective, and considerable efforts are now being made to provide a consistent theoretical description of the same. What motivates those efforts is the fact that the biological properties of molecules are directly conditioned by the changes in their electronic structure. These can often be fairly extensive, as evidenced by significant spectral shifts observed with the use of both stationary and time-resolved fluorescence spectroscopy. The existence of the phenomenon as such has, by now, been conclusively confirmed by researchers, and studies are now being conducted with the aim of identifying new molecules (natural as well as synthetic) that produce it. What has yet to be developed, however, is a conclusive and consistent theoretical explanation for the same (the most important theories that have been proposed in this context are discussed further in the text).

**Scheme 1:**
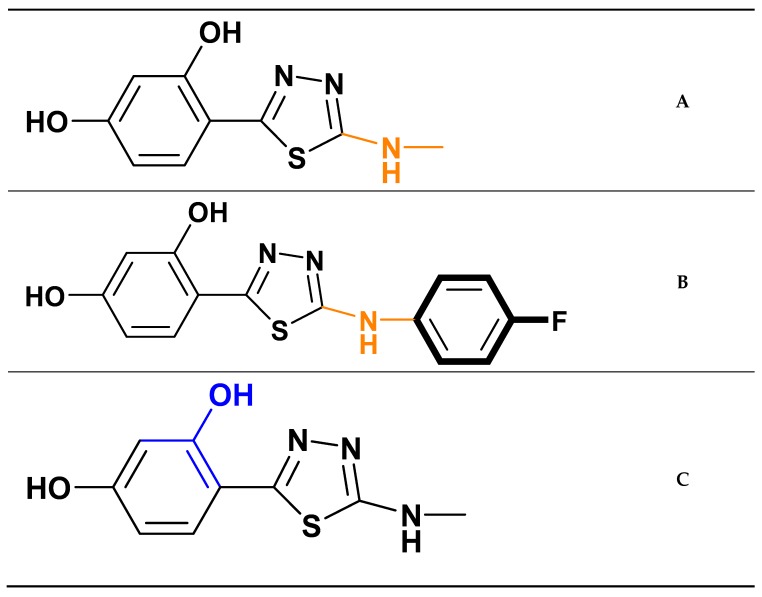

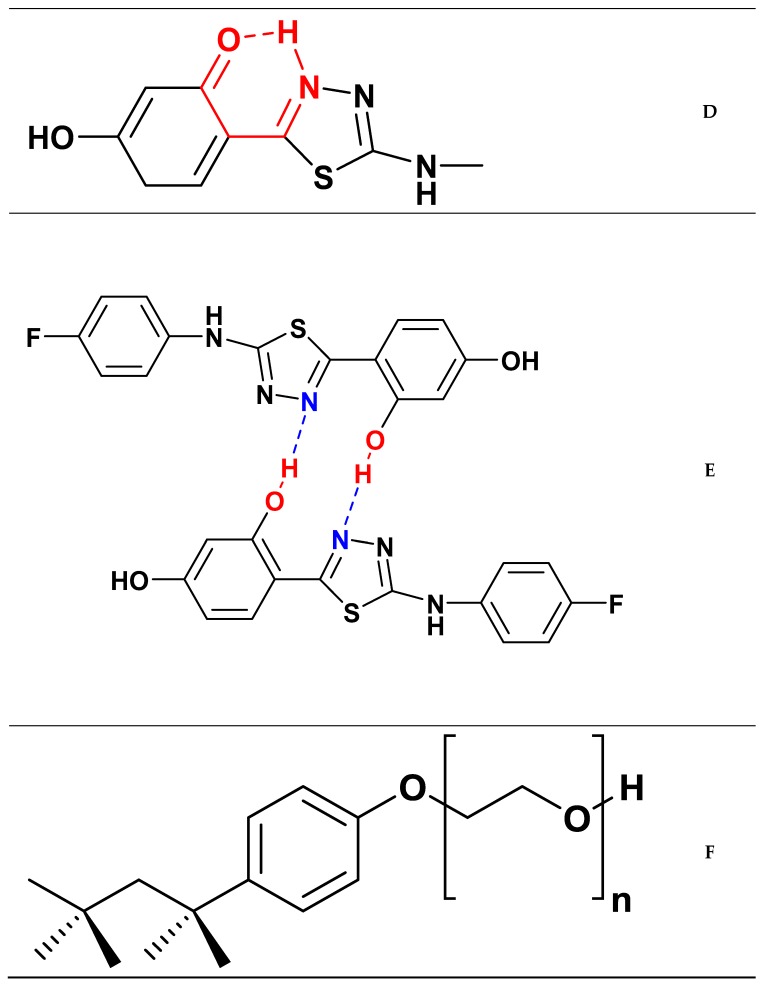
Panel (**A**)—General chemical structure of investigated 1,3,4-thiadiazoles. Panel (**B**)—Structure of FABT in neutral form. Panel (**C**)—The possible enol-form of analogues tested occuring in polar solvents, Panel (**D**)—The possibile keto-form of analogues tested occuring in non-polar solvents, Panel (**E**)—the most probable dimeric form occurring in the micellar system (the hydrogen bonds are marked in colour), Panel (**F**)—The chemical structure of Triton X-100.

An interesting group of compounds wherein inconsistencies with certain well established theories can be observed is the group of 2-amino-1,3,4-thiadiazoles substituted with the 2,4-dihydroxyphenyl system (Scheme 1A) [4].

From the pharmacological point of view, the analysed 1,3,4-tiadiazoles [5,6] substituted with the resorcylic fragment have been observed to have promising anticancer [7,8,9,10] and neuroprotective [11,12,13], anti-oxidative [7], antifungal and antibacterial activities [14,15,16,17], anti-inflammatory [18]. They also show certain very interesting properties as a group of ligands capable of forming complexes with selected D-block metals [6].

The compound selected for the presented spectroscopic study of the mechanisms of molecular interaction in a micellar Triton X-100 system was, due to its high bioactivity, 2-(4-fluorophenylamino)-5-(2,4-dihydroxybenzeno)-1,3,4-thiadiazole (Scheme 1B).

Apart from the aforementioned pharmacological properties, FABT is also characterised by very interesting spectroscopic effects, e.g., the effect of keto-enol tautomerism induced by changes in medium polarizability [19] as well as the effects of crystal polymorphism and solvatomorphism [20] and effects on lipid membranes entailing significant influence on their dynamics [21]. Moreover, other analysed analogues have been demonstrated to show a very interesting dual fluorescence effect induced, among other factors, by changes in the pH, concentration and temperature of the medium [4]. The ability to correlate the spectroscopic effects presented in the aforementioned papers with structural transformations of the analysed molecules could play a key role in explaining the pharmacological efficacy of the fluorophores (as well as the entire group of substituted 2-amino-1,3,4-thiadiazoles).

The aim of the present study was to perform a spectroscopic analysis of the molecular organisation of FABT (Scheme 1) in micellar systems. An attempt was also made to explain the observed fluorescence effects and their relation to a specific molecular form of the analysed fluorophore in the selected micellar system. Using spectroscopic methods such as: spectroscopy of electronic absorption and fluorescence, fluorescence lifetimes (TCSPC), the dynamic light scattering method (DLS), and chemometric analysis (PCA), we were able to demonstrate the complexity of the physical phenomena responsible for the phenomenon of dual emission observed for FABT molecules in a micellar environment.

Available literature describes the phenomenon of dual fluorescence [22] as related to the emergence of two clearly separated emission bands [23]. In which are induced by changes in e.g., the solvent’s polarity, medium’s pH [24], temperature [25], of the compound’s concentration. The most popular theories explaining the mentioned fluorescence effects include: the emergence of intramolecular CT states [26] often involving molecular twist i.e., the so-called Twisted Intramolecular Charge Transfer (TICT) states [27,28,29,30,31], molecular tautomerism emerging in excited states, related to the so-called excited-state proton transfer, i.e., Excited-state Intramolecular Proton Transfer (ESIPT) processes [32,33,34]. Furthermore, the effect can be induced by an increase of the compound’s concentration (emission originating from excimers (hetero- or homo-) [22] and processes related to breaking the Kasha’s rule (the most recent papers by Brancato et al. [34]), which in turn may also be related to ESIPT and TICT mechanisms.

Modelling the activity of fluorophores with the use of surfactants capable of forming micellar systems becomes a very important task. Various methods of conducting fluorescence measurements based on the changing dynamics of fluorophore distribution in protein films or membranes revealed a number of interesting qualities of the new fluorosensors such as e.g., fluorescein isothiocyanate (FITC—providing an excellent fluorescence probe against malicious ovary cancer cells which are characterised by high levels of folic acid expression) [35]. Other fluorosensors include e.g., 6-methoxyquinoline (6-MQ), a probe characterised by high sensitivity to chloride ions, used in studies on the ions’ transport through cell membranes, and additionally very easily located in the cells of living organisms [36]. Given the above uses, it is very important to model the behaviour of various fluorophores in micellar systems. In the context of such studies, one of the most important scientific concerns is the aforementioned effect of dual fluorescence observed in micellar systems.

In the case of the 1,3,4-thiadiazole selected for the study, the discussed fluorescence effects observed in micellar environments may be due to molecular aggregation closely related to the structure of the substituent system. In a particular case, the same can also generate CT related processes within the molecule (as well as in selected analogues in this group of compounds). Studies entailing the use, among other methods, of both stationary and excited state spectroscopy of the selected 1,3,4-thiadiazole analogue, facilitated the observation in the micellar environment of the effect of dual fluorescence (in certain concentration ranges of the compound itself as well as the selected detergent). This effect for this particular molecule dissolved in polar solvents has not been observed in any other study conducted to date. Learning the particulars of the analysed compound’s molecular organisation in the micellar environment may prove significant in evaluating its (as well as the whole group’s) potential pharmacological applicability and help to determine the directions of future modelling studies. Moreover, the molecule can be employed as a molecular probe highly sensitive to environmental changes in protein systems which, as already mentioned above, continue to be in exceptionally high demand in both photophysics and molecular biology. The commonly accepted CMC value for Triton X-100 in water is 0.2–0.3 mM, although some authors obtained slightly higher values when using different methods for the determination of the critical micelle concentration. In the paper [37] the CMC for Triton X-100 in water and cyclodextrin was determined using the RLS method which yielded the results of 0.5 mM and 1.1 mM, respectively. Due to the above, the CMC concentration was used in the present study at 0.4 mM (CMC). Furthermore double of the same (2× CMC) was used as well as concentration before reaching the CMC (BR-CMC).

## 2. Material and Methods

### 2.1. Materials

2-(4-Fluorophenylamino)-5-(2,4-dihydroxybenzeno)-1,3,4-thiadiazole (FABT, Scheme 1B) was synthesized in the Department of Chemistry of the University of Life Sciences in Lublin; details of the procedure are described elsewhere [38]. The structure of FABT is presented in the Cambridge Crystallographic Data Centre with entries: CCDC 768785–768787. Non-ionic surfactants Triton X-100 and phosphate buffer saline (PBS) were purchased from Sigma Aldrich (St. Louis, MO, USA) and used as received. For all experiments double distilled water used.

### 2.2. Methods

Triton X-100 stock solution (2.4 × 10^−2^ mol L^−1^) was prepared by dissolving 1.47 g of Triton X-100 reagent in water and diluting to the mark in a 100 mL calibrated flask. The operating solution of Triton X-100 was prepared by diluting the stock solution with water. Micellar systems were prepared by mixing the right amount of detergent and buffer. The detergent volume at the appropriate concentration was set at 10, 50 or 100 μL per 3 mL buffer, produced Triton concentrations of 0.08 mM—(before reaching the CMC (BR-CMC), 0.4 mM (CMC) and 0.8 mM (2× CMC), respectively. FABT was dissolved in methanol to give a starting concentration of 3 × 10^−3^ M. The volume of the FABT solution at the given concentration was set at 10, 20, 30, 40, 50, 60, 70 μL, per 3 mL of buffer, and produced concentrations of 0.01, 0.02, 0.03, 0.04, 0.05, 0.06, 0.07 mM, respectively. Measurements were made after 1 h from the preparation of micellar systems solutions.

### 2.3. Measurements of Electronic Absorption and Fluorescence Spectra

Electronic absorption spectra of FABT in the micellar system with Triton X-100 were recorded using a Cary 300 Bio double-beam UV-Vis spectrophotometer (Varian, Santa Clara, CA, USA) equipped with a thermostatted cuvette holder with a 6 × 6 multicell Peltier block. Temperature was controlled with a Cary Series II thermocouple probe (from Varian) placed directly in the sample. Fluorescence excitation, emission, and synchronous spectra were recorded with a Cary Eclipse spectrofluorometer (Varian) at 22 °C. Fluorescence spectra were recorded with 0.5 nm resolution and corrected for the lamp and photomultiplier spectral characteristics. Resonance light scattering measurements were performed as suggested by Pasternack and Collings [39]. The excitation and emission monochromators of the spectrofluorometer were scanned synchronously (no interval between excitation and emission wavelengths); the slits were set to obtain the spectral resolution of 1.5 nm. The spectral analysis was performed with the use of Grams/AI 8.0 software (Thermo Electron Corporation, (Waltham, MA, USA).

### 2.4. Time-Correlated Single Photon Counting (Tcspc)

Time-correlated single photon counting (TCSPC) measurements were performed using a FluoroCube fluorometer (Horiba, Longjumeau, France). The samples were excited with a pulsed NanoLED diode (Jobin Yvon IBH, Glasgow, UK) at 372 nm (pulse duration of 150 ps) operated with 1 MHz repetition. To avoid pulse pile-up, the power of the pulses was adjusted to an appropriate level using a neutral gradient filter. Fluorescence emission was recorded using a picosecond detector TBX-04 (Jobin Yvon IBH, Glasgow, UK). The DataStation and DAS6 software (Jobin Yvon IBH, Glasgow, UK)) were used for data acquisition and signal analysis. All fluorescence decays were measured in a 10 × 10 mm quartz cuvette, using an emitter cut-off filter with transmittance for wavelengths longer than 408 nm. The excitation profiles required for the deconvolution analysis were measured without the emitter filters using a light scattering cuvette. All measurements were performed in water at 22 °C and in two concentrations of, respectively, Triton X-100-10 and 100 μL (a given amount of μL Triton X-100 means a suitable concentration of detergent, as shown in the materials and methods). For both measurements series of FABT concentrations were used. Each fluorescence decay was analysed with a multiexponential model provided by Equation (1):

(1) $$\;I_{t}=\underset{i}{\sum }\alpha_{i}\exp\left(-t\tau_{i}\right)\;$$

where *α_i_* and *τ_i_* are the pre-exponential factor and the decay time of component *i*, respectively.

Best-fit parameters were obtained by minimization of the reduced χ^2^ value as well as residual distribution of the experimental data. The fractional contribution (*f_i_*) of each decay time and the average lifetime of fluorescence decay (<*τ*>) were calculated according to the following Equations (2) and (3):

(2) $$f_{i}=\frac{\alpha_{i}\tau_{i}}{\sum \alpha_{j}\tau_{j}}\;$$

(3) $$<\tau >=\sum f_{i}\tau_{i}$$

### 2.5. DLS Methods

The size of micelles was determined at 22 °C using Zetasizer Nano (Malvern Instruments Ltd., Worcestershire, UK) equipped with a laser (633 nm) set at an angle of 173°.

### 2.6. Statistical Treatment of Data. Principal Component Analysis (PCA)

PCA is a well-known multivariate statistical technique commonly used to analyse the nature of multivariate data representing the original dataset in the new orthogonal variables called principal components (PCs) [40]. The result of this method is a description of the originally considered dataset (correlated variables) by a lower number of (uncorrelated) variables. The new variables, PCs, are built as linear combinations of the old variables represented by the matrix *X*:

*PC_i_* = ∑*_i,j_a_ij_X_j_* (4)

This means that the data matrix *X* is represented as a product of two matrices, which are related by the formula *X* = SL^T^, where S and L denote the scores and loading matrices, respectively. The groups of the samples showing similar or different behaviours and characteristics are distinguished by the graphical representation of the scores. The first principal component explains the maximum amount of variance available in the original dataset. 

Principal Component Analyses (PCA) of fluorescence spectra were performed using OriginPro 2018 software (OriginLab, Northampton, MA, USA) using the Principal Component Analysis for Spectroscopy App.

## 3. Results and Discussion

Scheme 1A presents the structures of the studied 1,3,4-thiadiazole molecules with the 2,4-dihydroxyphenyl substituent. Scheme 1B presents the structure of 2-(4-fluorophenyl-amino)-5-(2,4-dihydroxybenzeno)-1,3,4-thiadiazole (FABT) in enol form. The conformation of the FABT molecule facilitates the emergence of the intramolecular hydrogen bond between the hydroxylic group in the *ortho* position from the resorcinyl ring and the nearest nitrogen atom from the 1,3,4-thiadiazole ring (Scheme 1). As mentioned above, the studied compound contains the characteristic 1,3,4-thiadiazole ring which, in the case of the FABT molecule, bonds to a secondary amine (–NH– group) and next to fluorobenzene as a substituent which can have a significant influence on the polarity of the compound selected for the study as well as its phase divisions.

### 3.1. Studies of Spectroscopic Effects of FABT in Micelle Systems

**Figure 1:**
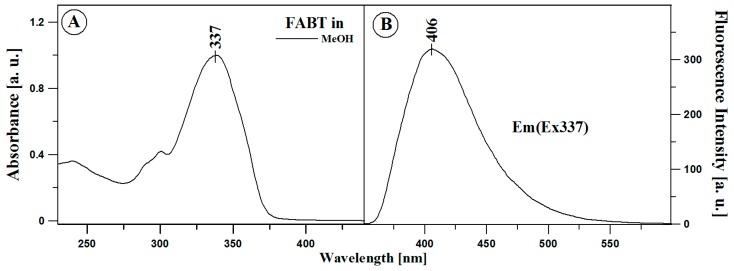
Panel (**A**)—Normalized electronic absorption spectra for FABT (Panel (**A**)) obtained in MeOH presented in the spectral range of 230–440 nm. Panel (**B**) presents fluorescence emission spectra of FABT recorded from MeOH. The emission spectra correspond to the measurements presented in Panel (**A**). The symbol Em(Ex337) stands for excitation emission spectra at 337 nm. The measurements were performed at the temperature of 22 °C.

Figure 1 (in Panel A) presents the electronic absorption spectra for FABT as measured in methanol (MeOH) (typical also for other polar solvents). The FABT absorption spectrum reveals a wide band stretching from approx. 300 nm to 375 nm with the maximum at 337 nm, which is characteristic of π→π* electronic transition. In the case of this compound a widening of the band on the long-wave side can be observed (with lower absorption and maximum at approx. 363 nm). The intensity of this widening (significantly lower than the one observed at 337 nm) and the relevant bathochromic shift confirm the possibility of the presence of aggregated forms in the samples (such as dimers, *N*-aggregates, Figure 1, Panel A) [41]. Panel B in Figure 1 presents the typical electronic fluorescence spectrum for FABT in MeOH, corresponding to the adequate absorption spectrum presented in Panel A. The excitation wavelength for the fluorescence spectrum corresponded to the maximum of the FABT absorption spectrum. For the presented fluorescence emission spectrum for FABT in MeOH, we observed a single emission bad with the maximum at approx. 406 nm.

**Figure 2:**
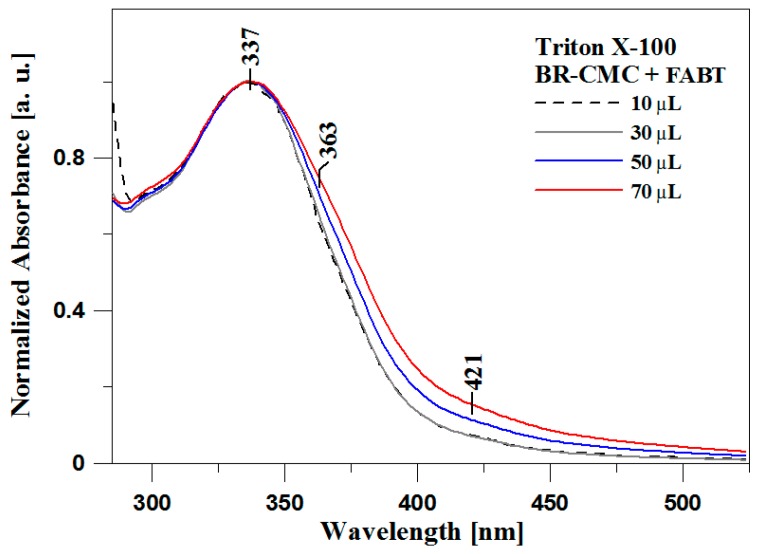
Normalized electronic absorption spectra for various amounts of FABT added in MeOH to the system with Triton X-100 detergent in the amount of 10 μL (BR-CMC) per 3 mL of the buffer. The measurements were performed at the temperature of 22 °C.

Figure 2 presents examples of normalised electronic absorption spectra for FABT in various concentrations of the compound in the micellar system formed with the detergent selected for the study. The amount of detergent used was 10, 50 or 100 μL per 3 mL of buffer, thus yielding Triton concentrations of 0.08 mM (BR-CMC), 0.4 mM (CMC), and 0.8 mM (2× CMC). The micellar system was titrated with varying amounts of FABT dissolved in MeOH in the concentration of c = 3.0 × 10^−3^ M. The figure presents the respective amounts of 10, 30, 50, and 70 μL of FABT used for titrating the detergent in the amount of 10 μL per 3 mL of the buffer. Adequate figures for the 50 and 100 μL detergent were presented in Figures S5 and S6 included in the Supplementary Materials. As can be observed in Figure 2 for FABT in the micellar system, similarly to Figure 1, the characteristic absorption band was present with the maximum approx. at 337 nm, which (as already mentioned above) is characteristic of the π→π* electronic shift in the studied 1,3,4-thiadiazole molecule. 

Relative to the changes in FABT concentration, we observed considerable changes in the shape of the registered spectra. For all the presented absorption spectra, we observed a significant widening of the absorption spectrum on the long-wave side with the maximum at approx. 363 nm, as well as an increase of intensity of the band with the maximum at approx. 421 nm. Both of these effect clearly evidenced (in accordance with Kasha’s Exciton Splitting theory [41]) the possibility of the presence in the analysed samples of non-monomeric forms of the compound (e.g., dimers or *N*-aggregates) [41]. For samples with Triton X-100 in the amount of 50 and 100 μL presented in the SM (Figures S5 and S6) we could observe similar correlations, although due to differences in the distribution of micelle size the spectra showed slightly higher dispersion (to be discussed further in the text).

**Figure 3:**
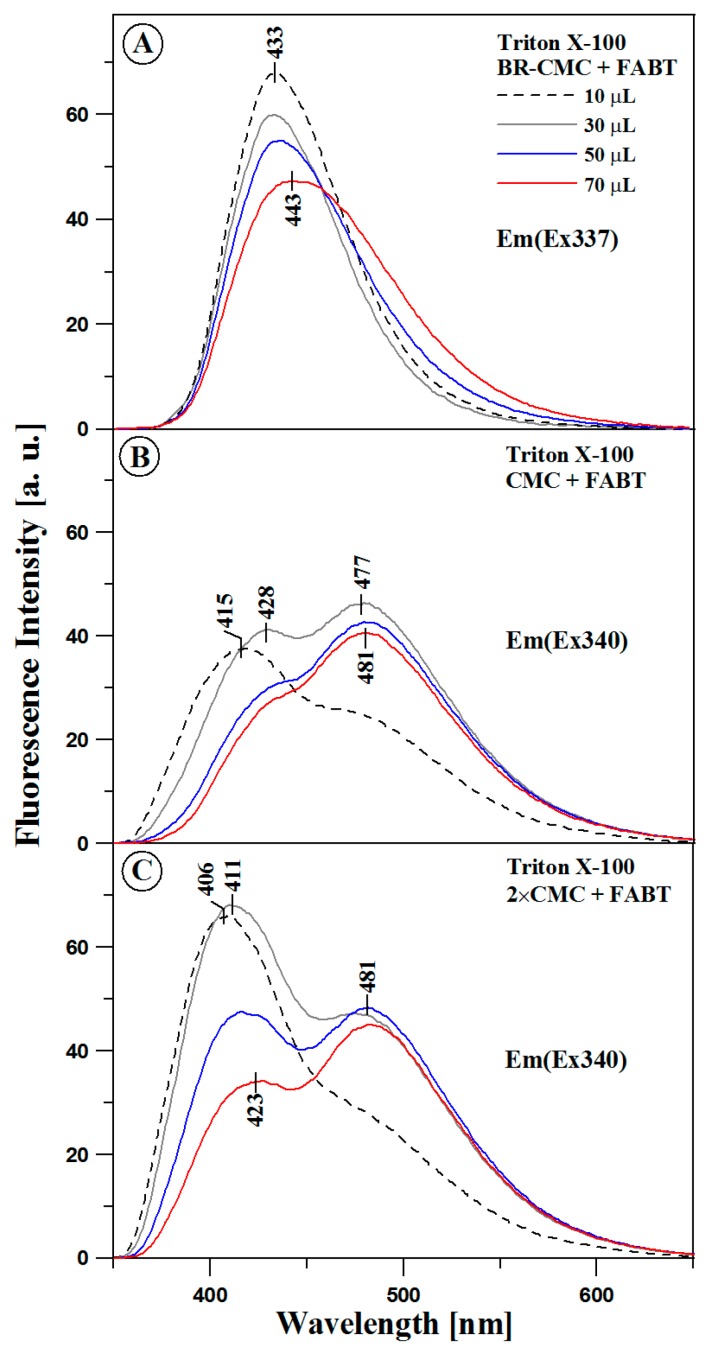
FABT fluorescence emission spectra corresponding to various concentrations of Triton X-100 per 3 mL of the buffer with subsequently titrated amounts of FABT at the concentration of c = 3 × 10^−3^ M. The spectra correspond to the measurements presented in Figure 2 and Figures S5 and S6 in Supplementary Materials. Panel (**A**) presents the results of the experiment conducted for the Triton X-100 in the amount of 10 μL per 3 mL of the buffer (BR-CMC), Panel (**B**) for 50 μL (CMC) of and Panel (**C**) for 100 μL (2× CMC) of the detergent. The symbol Em(Ex337/340) stands for excitation emission at the corresponding wavelength, the rest analogically. The spectra were recorded at the temperature of 22 °C.

**Figure 4:**
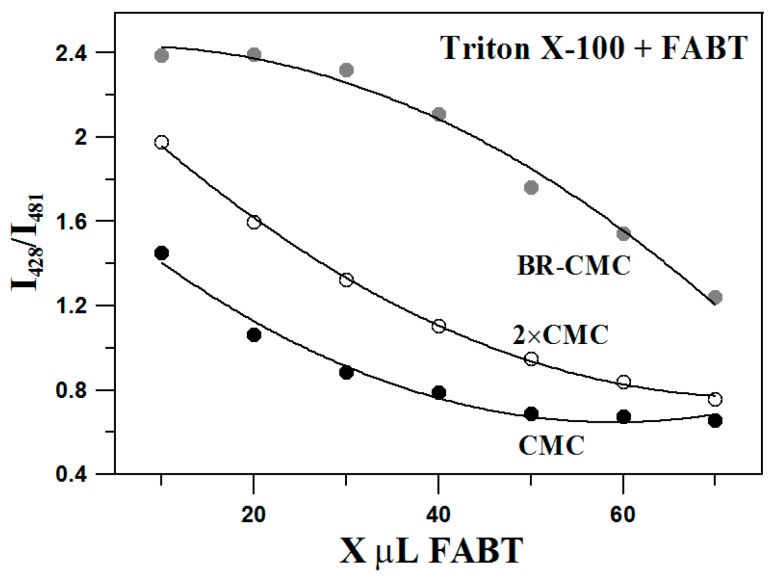
Correlations between the intensity of fluorescence at the wavelength of 428 to 481 nm and changes in the amount of FABT added analogously to Figure 3. Points of measurement were matched with polynomial curves.

Particular attention should be paid to the effect presented in Panels A, B and C of Figure 3 depicting the florescence emission spectra corresponding to the respective electronic absorption spectra in Figure 2 (as well as Figures S1 and S2). The excitation wavelength in all samples corresponds to the maxima of the absorption spectra, respectively 337 nm in Panel A and 340 (337) nm in Panels B and C. As can be observed in Panels A–C, depending on the concentration of the compound (FABT) in the micellar system, we observed the presence of the dual fluorescence effect conditional upon the amount of the detergent and, above all, the concentration of FABT. In Panel A, where spectra of fluorescence emission for various FABT concentrations in Triton X-100 were presented, we observed a single emission band with the maximum at 433 nm which, as the compound concentration increases, shifts in the long-wave direction to approx. 443 nm (in particular for approx. 70 μL of FABT in the detergent system—red line in Panel A). Additionally, the band is noticeably widened, which suggests the possibility of aggregated forms, e.g., dimers, in the micellar system. The results are consistent with previous studies performed on other analogues from the same group of compounds[4]. In the first phase of the experiment, i.e., in the system prior to CMC (BR-CMC), the concentration of the detergent was too low to allow the formation of micelles or the number of micelles was too small to effectively observe aggregative activity in the analysed system, most of the added FABT molecules could remain in their monomeric form. Therefore, before the shift of the emission spectrum, its significant widening, and the decrease in its intensity (often characteristically accompanying aggregative activity), we observed no other effects. Subsequently, as the concentration of the detergent molecules increased, inter-molecular interactions led to the aggregation of detergent molecules, and in effect, once CMC was reached, formation of micelles. With increasing FABT concentration in the system, we observed a very interesting effect of dual fluorescence. As can be seen in in Panel B of Figure 3, as soon as the FABT content reached 10 μL, we started to observe a band with the emission maximum at approx. 415 nm (characteristic of monomeric forms) as well as dual fluorescence emission with the maximum at approx. 470 nm (characteristic of dimeric forms or even larger *N*-aggregates of the compound). As the concentration of the compound increased while the value for the detergent remained constant, we observed a solvatochromic-bathochromic shift of the band with the maximum at 415 to 428 nm as well as a significant increase in the intensity of the band with the maximum at 470 nm (and its shift to 477 nm). The observed spectral changes clearly evidence an increase in the number of aggregated forms in the analysed system and formation of aggregative forms in the respective micellar systems with Triton X-100. With a significant increase in the concentration of FABT in the detergent, we observed a significant decrease in the intensity of the 428 nm band accompanied by an increase in the intensity of the band with the maximum at 481 nm (connected with a shift of the band with the maximum at 477 nm). In this system and for this concentration, we observed a significant prevalence of aggregated forms of FABT in the micellar system, which may have included dimer type aggregates as well as progressively larger, *N*-aggregated forms. Panel C in Figure 3 presents the fluorescence emission spectra for the fixed amount of the Triton X-100 detergent necessary to reach 2× CMC (i.e., concentration with a change in FABT concentration analogous to that observed previously (in Panels B and C). The observed effects were similar to those in Panel B but one should emphasise the significantly stronger quenching effect with regard to the two bands, which evidences a much greater impact of aggregative processes in the observed dual emission effect. Additionally, even for a high FABT concentration in the micellar system, we observed clear bands with the maxima at, respectively, 423 and 481 nm, with the band of the first emission clearly losing intensity with increasing FABT concentration while the intensity of the long-wave emission was simultaneously significantly increased. Furthermore, we could also note that with increasing amount of the detergent, the band of the first emission was present in various locations, i.e., for 10 μL of the detergent (Triton X-100) it was observed for low FABT concentrations at approx. 433 nm, while as the detergent content increased, it shifted towards the short-wave side, as far as approx. 423 nm (Figure 3, Panels A and C). This could suggest that even in low Triton X-100 and FABT concentrations in the system, a large amount of dimeric forms was present (from previous studies on various analogues), which is clearly evidenced in Figure 2 (and Figures S1 and S2). As can be seen, the observed fluorescence effects depended both on the amount of the detergent used and the concentration of FABT in the micellar system. This suggests effects related to chromophore aggregation of the compound within the system, which was here observed in a model biological, micellar system. The aforementioned effects are particularly well visible in Figure 4 presenting in Panels A, B and C, for all systems in Figure 3, the correlation between the fluorescence emission intensity at the wavelength of 428 to 481 nm on the one hand, and the amount of FABT present in the given micellar system on the other. The figure shows an inverse relationship between the cases where single fluorescence emission spectra are observed relative to the case where the dual emission effect is seen.

**Figure 5:**
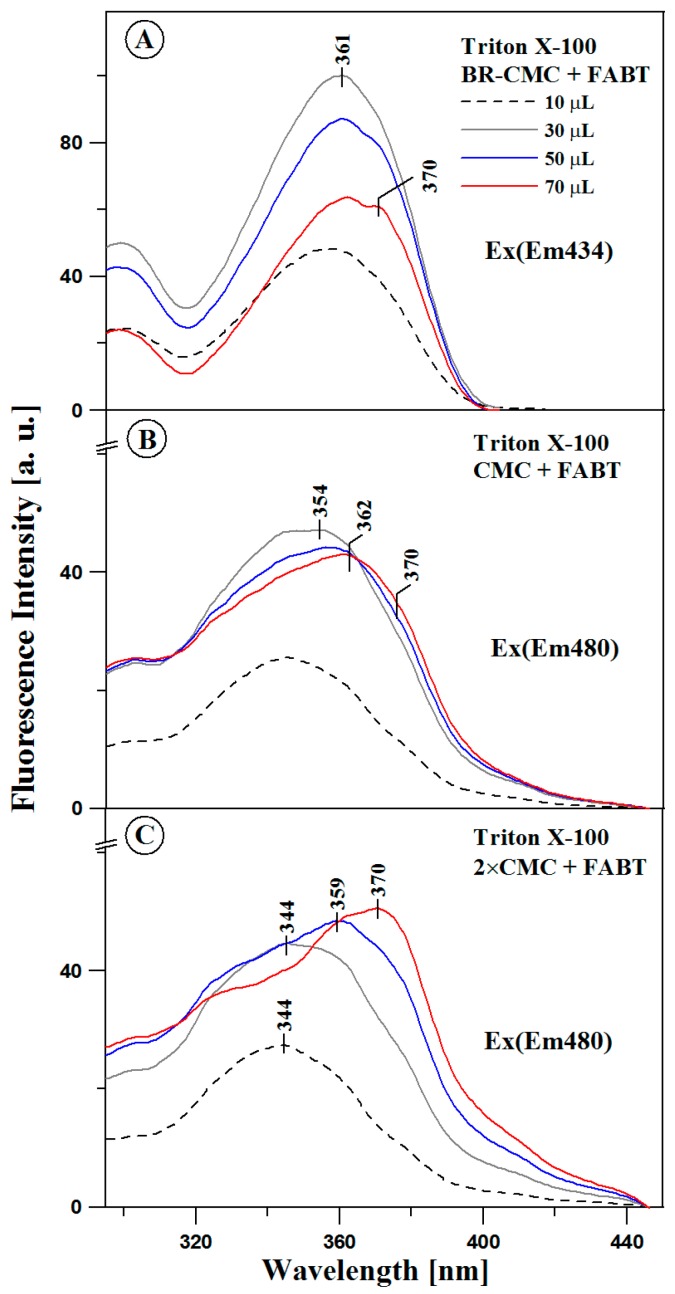
Fluorescence excitation spectra corresponding to emission spectra in Figure 3 (Panel A, B and C). The Ex(Em480) stands for excitation emission at 480 nm, the rest analogically. Panel (**A**) presents the results of the experiment conducted for the Triton X-100 in the amount of 10 μL per 3 mL of the buffer (BR-CMC), Panel (**B**) for 50 μL (CMC) of and Panel (**C**) for 100 μL (2× CMC) of the detergent. The spectra were recorded at the temperature of 22 °C.

Figure 5 presents the fluorescence excitation spectra (Ex) for FABT in the micellar system with Triton X-100, corresponding to the fluorescence emission spectra in Figure 3 (Panels A, B and C). Excitation emission was registered at the wavelength of 434 nm (for all FABT concentrations in Panel A) and 480 nm (for all concentrations in Panels B and C in Figure 5). A considerably greater selectiveness of fluorescence excitation spectra relative to electronic absorption spectra makes it possible to excite a particular molecular form of the compound in the studied system. In Panel A of Figure 5, with increasing compound concentration in the micellar system we practically always observed a minor strengthening on the long-wave side of the band with the maximum at approx. 370 nm. The same was the most apparent for 70 μL of FABT, which, as evidenced by the fluorescence emission spectra, corresponds to a significant widening and shift of the emission band and is associated with an increase in dimer-type aggregates in a “card pack” system. In Panel B this effect was considerably more visible, we observed a shift of the fluorescence excitation band from 354 to 362 nm, which corresponded to the increase of the compound’s concentration in the micellar system. Additionally, with the maximum at approximately 370 nm, we observed a significant strengthening of the band, which indicated the prevalence of aggregated dimers and/or *N*-aggregates. The effect was not so clearly visible in the absorption spectra obtained from the previous experiment. In Panel C, for the detergent concentration of 2× CMC, the aggregative effects became even more evident with the increasing compound concentration in the micellar system. At the initial stage of the experiment, we observed increased spectral intensity after adding FABT in the amount of 30 μL (as done previously in Panels A and C), followed by the subsequent quenching of the band characteristic of monomeric forms and a very significant strengthening of band intensity at 370 nm associated with the exciton splitting at the main energy level S_0_. Increased intensity of this band, combined with increased concentration of the compound in the micellar system should, in accordance with M. Kasha’s exciton splitting theory, be associated with strong prevalence of aggregated forms of FABT in the system. Furthermore, in Panel C at a wavelength of approximately 405 nm, we could also observe broadening of the band (reminiscent of the effect observed previously for absorption spectra) and the effect may also signify prevalence of larger aggregated systems (*N*-aggregates). Also in Panel C, for FABT in the amount of 70 μL, we observed a clear exciton splitting of the bands in the short-wave and long-wave direction, the former associated with the prevalence of dimers in the micellar system and the latter related to the presence of *N*-aggregated “head-to-tail” systems.

**Figure 6:**
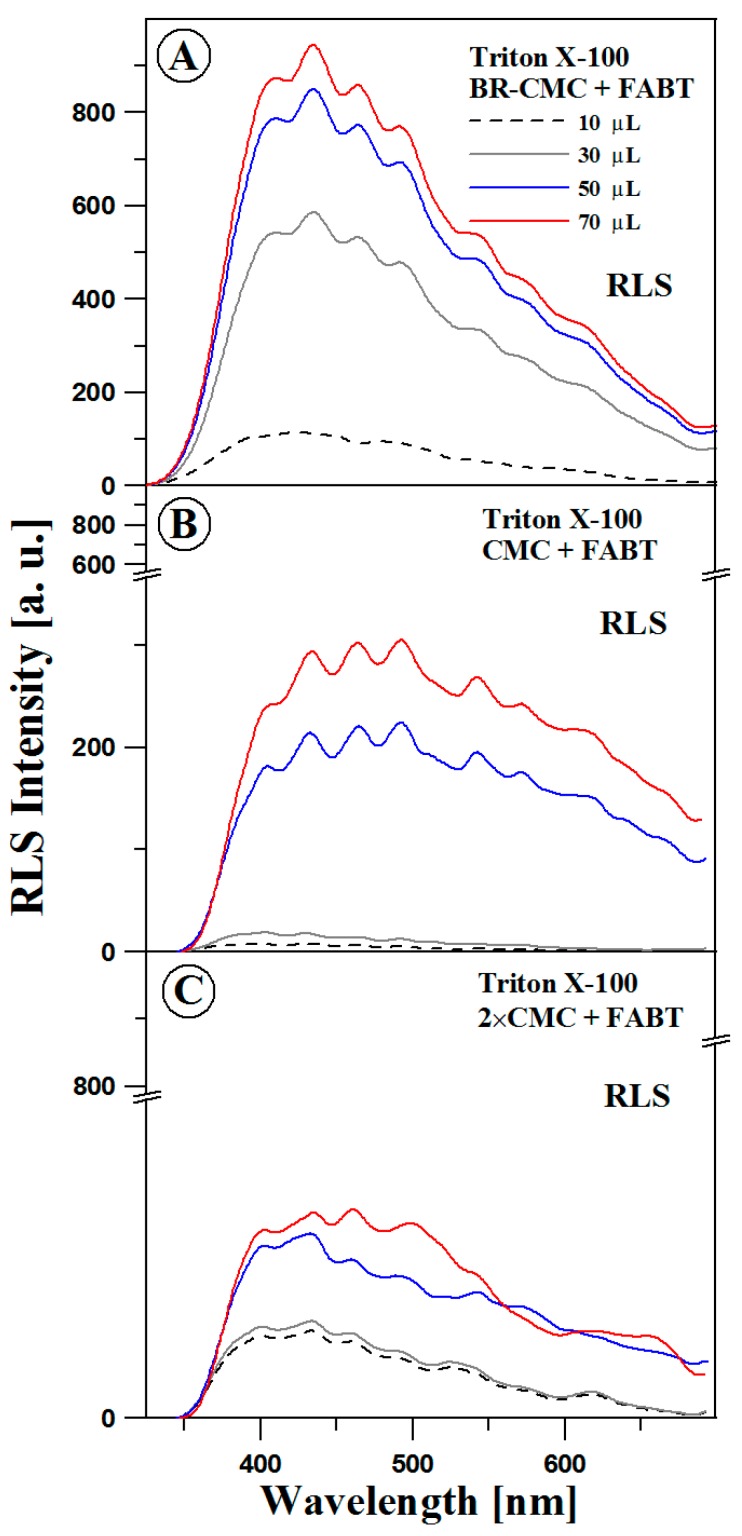
Spectra of resonance light scattering (RLS) in an experiment analogical to that in Figure 2, Figure 3, Figure 4, Figure 5, Figure 7, Figure 9 and Figure 10. The measurements were performed at the temperature of 22 °C.

**Figure 7:**
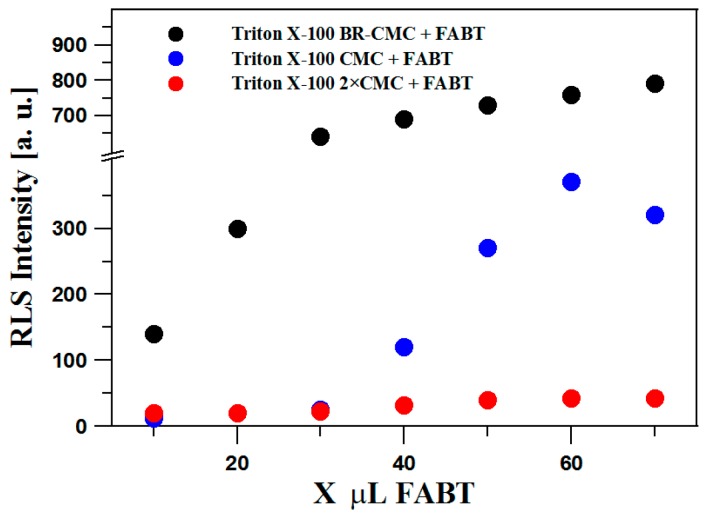
Correlation between the intensity of RLS spectra for the spectra presented in Figure 6 at the wavelength of 450 nm, depending on the amount of FABT added to the micellar system, analogically to previous experiments.

To recapitulate the results so far based on the exciton splitting theory, the characteristic long-wave bands should, in this case, be associated with various types of aggregated forms of the studied compounds, which is further evidenced by the RLS spectra (presented in Figure 6 and Figure 7). According to the aforementioned theory, the observed shifts are related to two forms of aggregation: the hypochromic shift corresponds to the formation of “card pack” aggregates while the bathochromic shift stems from “head to tail” aggregation [41]. Based on the above, it can be concluded that the observed fluorescence effects are clearly related to the effect of aggregation of FABT molecules in selected micellar systems [4,20,42] and indeed may be associated with Aggregation-induced emission (AIE) [43]. In contrary, our findings suggest that the aggregation can only favour the intermolecular CT processes, which in turn give rise to the dual emission in FABT. In the subsequent part of the study, DLS and RLS measurements were performed (as already explained above), which largely confirmed these assumptions (see below).

### 3.2. DLS Study

**Figure 8:**
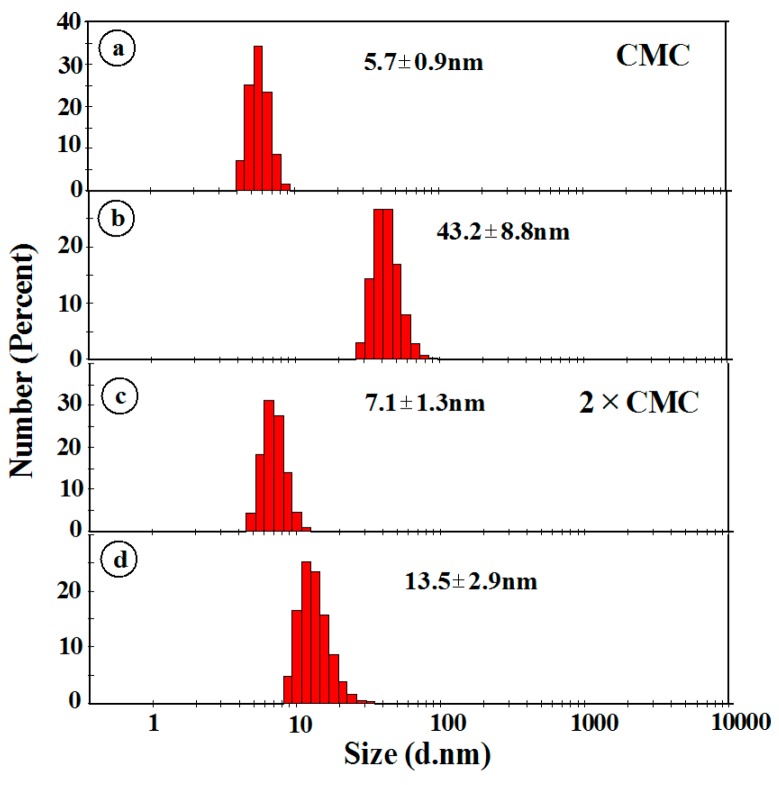
Size distribution histograms of the assemblies determined by DLS measurements carried out in PBS buffer solutions of (**a**) Triton X-100 CMC, (**b**) Triton X-100 CMC + FABT (0.07 mM), (**c**) Triton X-100 2× CMC, (**d**) Triton X-100 2× CMC + FABT (0.07 mM). The measurements were performed at the temperature of 22 °C.

DLS measurements were conducted with the view of determining the change in micelle hydrodynamic diameters for CMC, and 2× CMC, as well as the associated FABT adducts. It was revealed that the measured hydrodynamic diameters in CMC Triton X-100 were similar to values quoted in literature [44,45]. The hydrodynamic diameters (see Figure 8) for the aforementioned solutions were 5.7 ± 0.9 nm and 7.1 ± 1.3 nm. After the formation of the FABT-micelles adducts, were 43.2 ± 8.8 nm and 13.5 ± 2.9 nm for CMC and 2× CMC, respectively. Annotations indicate the mean size of diameter and standard deviations. This clearly shows that the micellar structures are enlarged after the incorporation of FABT molecules. Structures observed in the BR-CMC solution were large-diameter (~300 nm) (see Supplementary Materials) and most likely should not be related to the micellar structure. 

### 3.3. Resonance Light Scattering—RLS Study

**Figure 9:**
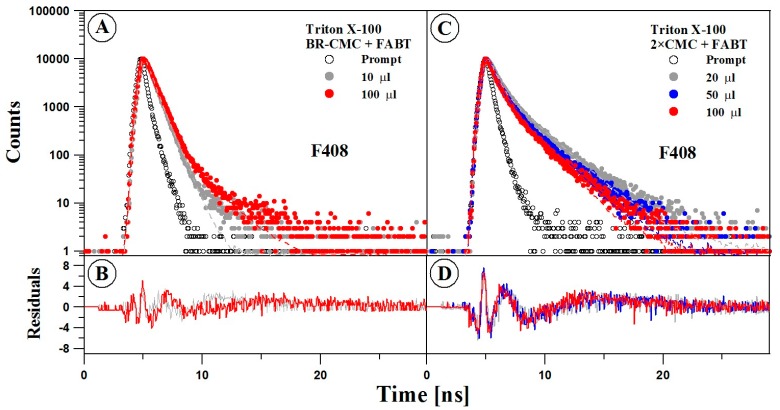
Effect of different FABT content in Triton X-100 (different concentrations) on the fluorescence decay of FABT (Panels (**A**,**C**)). In Panels (**A**,**C**), the dotted curves show the decay of FABT fluorescence emission observed using the TCSPC technique in the micellar system, while continuous lines are double exponential fits (grey—10 μL of FABT (BR-CMC), red—100 μL of FABT (2× CMC), blue—50 μL (CMC) of FABT in all panels). Panels (**B**,**D**) show residual plots (determined for the data in Panels (**A**,**C**)). The excitation pulse profile set up at 372 is indicated by the black dotted curve. The measurements were performed at the temperature of 22 °C.

**Figure 10:**
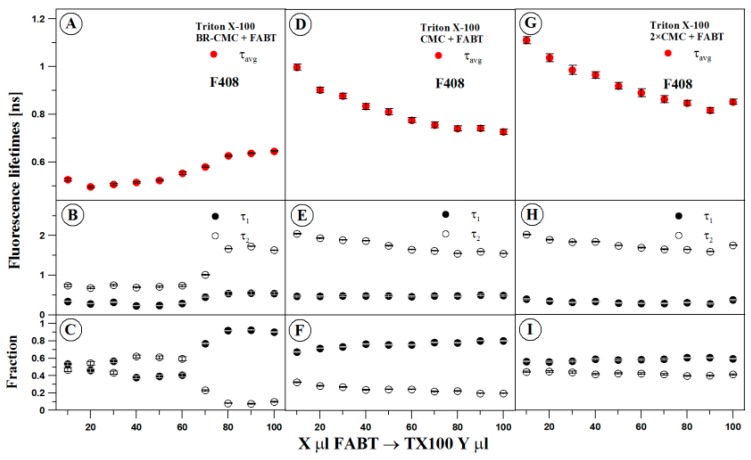
Correlation between fluorescence lifetimes and the amount of FABT added to the aforementioned amounts of detergent. Panels (**A**,**D**,**G**) present average fluorescence lifetimes observed for various amounts of Triton X-100. Panels (**B**,**E**,**H**) show fluorescence lifetimes of the first (*τ*_1_) and the second (*τ*_2_) component of FABT, observed for various concentrations of FABT in Triton X-100. The dependences of the fraction of both components on FABT in Triton X-100 values are presented in Panels (**C**,**F**.**I**). Panel (**C**) presents the results of the experiment conducted for the Triton X-100 in the amount of 10 μL per 3 mL of the buffer (BR-CMC), Panel (**F**) for 50 μL (CMC) of and Panel I for 100 μL (2× CMC) of the detergent. The data analysis was carried out for the two components model (Equation (1) for *i* = 2) for data presented in Figure 9. The spectra were recorded at the temperature of 22 °C.

RLS spectra for FABT in solutions analogous to those described above (Figure 2, Figure 3, Figure 4, Figure 5, Figure 9 and Figure 10) were measured with the view of verifying and confirming the impact of aggregation on the observed fluorescence effects. Figure 7 presents the correlation between RLS spectrum intensity and the amount of FABT in the studied micellar system. Similarly to previous figures the black dots correspond to the amount of detergent in BR-CMC, blue dots to CMC, and red dots to 2× CMC. As can be somewhat surprisingly observed (although similar effects were already mentioned in the case of measurements of electronic absorption spectra—in Figure 2 and Figures S5 and S6), the highest intensity of RLS bands was obtained in the case of the BR-CMC system (a result already suggested by the DLS measurements). For CMC, the intensity increase was significantly less pronounced and became clearly visible only after the addition of 40 μL of FABT into the solution. The weakest RLS band strengthening was observed for the largest amount of detergent in the micellar system, i.e., for 2× CMC, and corresponded to the increase of FABT concentration in the system. The same is illustrated in Figure 6, where Panels A-C present the RLS spectra (Δλ = 0) for FABT, from which the relevant values presented in Figure 7 were derived. As observed by Parkash [39] the presence of RLS bands should be associated with chromophoric aggregation occurring in the solution [39]. Because RLS spectra for pure Triton X-100 are observed at the wavelength of approx. 280 nm [37] and the RLS signal for pure FABT is characterised by very low intensity, it should be assumed that the aforementioned RLS bands are characteristic of FABT—Triton X-100 systems. Once the above results are confronted with the DLS measurements, certain very interesting correlations can be observed. The DLS measurements in the BR-CMC + FABT system revealed aggregated structures of relatively large size, approx. 1000 nm, (see Supplementary Materials) which corresponded to the highest intensity of the RLS band in this system. 

For CMC and 2× CMC, the RLS intensity was significantly lower. The results indicate that the RLS signal in the analysed systems originates mainly from aggregated structures with a large hydrodynamic diameter. On the other hand, the ratio of RLS band intensity at 440 nm to 480 nm for BR-CMC is different from that observed for CMC and 2× CMC. This suggests that analysis of RLS bands can be used to monitor FABT aggregation in micellar systems. As can be further observed, changes in the FABT concentration in the micellar system correspond to the presence in RLS bands of several bands evidencing the diversity of aggregated structures (such as dimers or larger *N*-aggregates). 

To sum up the RLS results it should be pointed out that RLS related to chromophoric aggregation of FABT molecules are very characteristic and clearly link the observed fluorescence effects to the chromophoric aggregation of the studied compounds. The oscillative structure of the observed RLS bands evidences the diversity of aggregated structures of different sizes [39]. Furthermore, RLS spectra clearly suggest (as already mentioned above in the description of the results obtained from DLS measurements) that whether the effect of duality of FABT emissions can be observed depends on the distribution of micelle sizes in the studied micellar system.

### 3.4. Fluorescence Lifetimes Study 

Measurements of fluorescence lifetime (TCSPC) were also performed for FABT in the analysed micellar systems, specifically a system with the Triton X-100 concentration below CMC (Figure 10, Panel A and Table 1) and above CMC (Figure 10, Panel B). An analysis of results obtained from deconvolution of 1, 2, and 3-component models revealed that in all considered cases, the best model was always the binary model. The unary model failed to properly reflect fluorescence decay, while the use of the ternary model did not yield sufficient quality improvement to be justified. In the first case, the increase in FABT concentration resulted in a clear extension of the fluorescence lifetime, while in the presence of micelles an inverse correlation was observed. Increased concentration of FABT correlated with reduced fluorescence lifetime. The presented average lifetime results (Panels A, D, and G in Figure 5) clearly suggest that in the case of Panel A (Figure 5), where we observed prevalence of monomerised forms (too few micellar systems), with the increasing concentration of FABT the average fluorescence lifetime was extended. Meanwhile, in Panels D and G (Figure 5), a significant reduction of the average fluorescence lifetime in FABT molecules was observed. In this case (as can be inferred from previously obtained results) we are dealing with aggregation of FABT molecules taking place in the micellar system. The process of aggregation induces the emergence of the dual fluorescence emission effect in this system. As already observed for other studied analogues from the 1,3,4-thiadiazole group in solvent systems, the process of aggregation often remains without a significant influence on the absorption spectra of the given analogue. However, it increases the possibility of charge transfer (CT) states, which in turn induces dual fluorescence emission effects for FABT in a micellar system. This is confirmed by the slight reduction of the average lifetime in the CMC and 2× CMC systems, as well as the fact that even when reduced, the same still remains, on average, significantly longer than in the case before CMC. The adequate conformation of FABT molecules (in systems above 2× CMC) with the –OH group from the resorcylic ring on the side of the nitrogen atom from the thiadiazole ring, allows the formation of a hydrogen bond between said groups. This system may suggest processes related to the possibility of charge transfer in this system, which in turn is most likely responsible for the effect of dual fluorescence observed in FABT molecules (to be further investigated in the subsequent publications in this series). This is corroborated by the average lifetime which is longer in pure solvent systems as well as by a number of accounts available in literature [4].

### 3.5. Principal Component Analysis (PCA)

In order to better compare data from different samples we applied the multivariate statistical analysis which is PCA. For this we recorded a set of 34 spectra of 2-(4-fluoro-phenylamino)-5-(2,4-dihydroxybenzeno)-1,3,4-thiadiazole*.* PCA was performed on a 34 × 276 matrix, where the rows represent the considered samples and the columns the variables, and included the fluorescence intensities for different points in the studied spectra. We considered fluorescence intensities in the region between 375 nm to 650 nm, which was selected due to the fact that it contains the maxima of all analysed samples. PCA values were obtained from a covariance matrix. The results of the PCA analysis of data are reported in Table S1. Figure S1 shows the contribution of the eigenvalues with principal components. As we can see, the first three principal components have a large contribution to the total eigenvalues, therefore, in our analysis we expressed the original data in the terms of this PC. The remaining components labelled with numbers greater than three would be noise factors, leading to background intensities.

3D plots (PC1 × PC2 × PC3) of scores and loadings from PCA applied to the considering data are shown in Figure S2a,b 99.84% of the total variance is explained in the first three PCs. PCA classification based on score plot is presented on Figure S2b. The spectrum name is explained as follows: TX *n*-*m_l_*(*k*) and TX *n*-*m*_2_(*k*) for *n*∈{50, 100}, *k*∈{340, 360}, *l* = 1, 2 denote Triton X-100 *n*-*m_l_* uL Em(Ex*k*), where *m*_1_∈{10, 20,…,100 } for *n* = 100 and *m*_1_∈{10, 20,…,70} for *n* = 50.

The spectra can be divided into three regions: Region I containing 7 spectra clustered with positive PC3 values, Region III containing 6 spectra for which PC1 takes positive values from the interval (30, 77) and negative values from the interval (−44, −29) for PC3. Four spectras: Triton X-100: 2× CMC + 10 µL FABT (Em 340 nm), 2× CMC + 30 µL FABT (Em 340 nm), 2× CMC + 10 µL FABT (Em 360 nm), CMC + 30 µL FABT (Em 340 nm) were excluded from the set of determined regions.

The plots of PCA loading factor vs. wavelength (Figure S3) allowed us to identify the spectral waveform of each PC. The nine fluorescence peaks P1(406 nm), P2 (411 nm), P3 (415 nm), P4 (423 nm), P5 (428 nm), P6 (433 nm), P7 (443 nm), P8 (477 nm), P9 (481 nm) can be describes in relation to the PCs. As we can notice for peaks P3–P7, all three PCs take negative values. The peak P7 can be identified as closely related to the minimum of PC3. The peaks P8 and P9 are related to the minimum of PC2.

## 4. Conclusions

The presented studies conducted with the use of fluorescence spectroscopy indicate the emergence of dual fluorescence in the studied compound from the 1,3,4-thiadiazole group in non-ionic micellar systems with Triton X-100. The fluorescence spectroscopy analyses, measurements of average fluorescence lifetimes and DLS, as well as the PCA analysis revealed a strong correlation between this effect and the process of molecular aggregation as well as the concentrations of the compound itself (aggregative capacity) and the detergent, with the latter determining the possibility of obtaining various micellar systems. The analyses of fluorescence emission spectra for FABT indicated the emergence of the dual fluorescence effect within the range of detergent concentrations corresponding at least to its CMC and 2× CMC, i.e., in the case in question, respectively: 0.4 mM and 0.8 mM. In concentrations lower than the CMC (BR-CMC), where the amount of the detergent was too low to form sufficiently many micelles, we observed a significant prevalence of monomeric forms of FABT and absence of the dual fluorescence effect. The detailed measurements of stationary and time-resolved fluorescence as well as results obtained with the use of other techniques mentioned above indicate that the effect of dual emission is also accompanied by significant quenching of fluorescence emission related to aggregation. Furthermore, despite the reduction of the average fluoresce lifetime within the experimental framework of CMC and 2× CMC, it nonetheless remains longer than that obtained in the experiment with a concentration lower than CMC (BR-CMC). This fact is a clear indication of the possibility of processes related to charge transfer (CT) in FABT molecules which, provided that the proper conformation of the molecule is maintained, are triggered by the processes of molecular aggregation. A significant impact on the observed effects related to the changing dynamics of the studied fluorophore is also dependent on the quantity of detergent, which determines the (mono- or polydispersive) qualities of the given system.

Based on the above studies, it will be possible to perform a quick analysis of the molecular organisation of the studied molecule as well as the entire 1,3,4-thiadiazole group in model systems of biological significance with the use of fluorescence methods. The FABT molecule itself may provide an ideal fluorosensors, which strengthens the validity of conducting further research into systems capable of micellization.

## Figures and Tables

**Table 1:** Fluorescence lifetimes in BR-CMC, CMC and 2× CMC samples.

Triton X-100	BR-CMC	CMC	2× CMC
+FABT	*τ* ± Δ*τ* [ns]	*τ* ± Δ*τ* [ns]	*τ* ± Δ*τ* [ns]
10	0.526 ± 0.006	0.986 ± 0.014	1.105 ± 0.016
20	0.496 ± 0.004	0.885 ± 0.013	1.027 ± 0.018
30	0.505 ± 0.005	0.859 ± 0.013	0.973 ± 0.020
40	0.515 ± 0.004	0.813 ± 0.013	0.951 ± 0.015
50	0.523 ± 0.003	0.790 ± 0.013	0.903 ± 0.015
60	0.553 ± 0.005	0.753 ± 0.014	0.873 ± 0.018
70	0.579 ± 0.003	0.732 ± 0.012	0.844 ± 0.016
80	0.626 ± 0.002	0.716 ± 0.011	0.828 ± 0.011
90	0.636 ± 0.002	0.717 ± 0.012	0.796 ± 0.012
100	0.645 ± 0.002	0.702 ± 0.012	0.833 ± 0.011

## Supplementary Materials

The following are available online, Figure S1: Contribution of eigenvalues with PCs, Figure S2: 3D plots (PC × PC2 × PC3) of loading (a) and score (b) from PCA, Figure S3: The loading vectors for PCs plotted as a function of wavelength. The plots indicate which spectral shape are associated with variance in the overall signal, Figure S4: Distribution of DLS intensities measured in PBS buffer solutions of (a) Triton X-100 BR-CMC (blue line), Triton X-100 BR-CMC + FABT (red line) (b) Triton X-100 CMC (blue line), Triton X-100 CMC + FABT (red line) (c) Triton X-100 2× CMC (blue line), Triton X-100 2× CMC + FABT (red line), Figure S5: Normalized electronic absorption spectra for various amounts of FABT added in MeOH to the system with Triton X-100 detergent in the amount of 50 μL per 3 mL of the buffer. The measurements were performed at the temperature of 23 °C, Figure S6: Normalized electronic absorption spectra for various amounts of FABT added in MeOH to the system with Triton X-100 detergent in the amount of 100 μL per 3 mL of the buffer. The measurements were performed at the temperature of 23 °C, Table S1: Results of Principal Component Analysis performed on dataset.
