# Supplementary material for: Spectroscopic Studies of Dual Fluorescence in 2-(4-Fluorophenylamino)-5-(2,4-dihydroxybenzeno)-1,3,4-thiadiazole: Effect of Molecular Aggregation in a Micellar System
Source: Molecules. 2018 Nov 2;23(11):2861. doi: 10.3390/molecules23112861 (PMC6278424; doi:10.3390/molecules23112861)
Supplement: Supplementary file 1 [file molecules-23-02861-s001.pdf]

molecules

Aticle

Spectroscopic Studies of Dual Fluorescence in 2-(4-Fluorophenylamino)-5-(2,4-dihydroxybenzeno)-1,3,4-thiadiazole: Effect of Molecular Aggregation in a Micellar System

Grzegorz Czernel [1], Arkadiusz Matwijczuk [1,*], Dariusz Karcz [2], Andrzej Górecki [3], Agnieszka Niemczynowicz [4], Aleksandra Szcześ [5], Grzegorz Gładyszewski [6], Alicja Matwijczuk [1], Bożena Gładyszewska [1] and Andrzej Niewiadomy [7,8]

1 Department of Biophysics, University of Life Sciences in Lublin, Akademicka 13, 20-950 Lublin, Poland; grzegorz.czernel@up.lublin.pl (G.C.); alicjakruk@vp.pl (A.M.); bozena.gladyszewska@up.lublin.pl (B.G.)
2 Department of Analytical Chemistry (C1), Faculty of Chemical Engineering and Technology, Krakow Technical University, Warszawska 24, 31-155 Krakow, Poland; dariuszkarcz@indy.chemia.pk.edu.pl
3 Department of Physical Biochemistry, Faculty of Biochemistry, Biophysics and Biotechnology of the Jagiellonian University, Gronostajowa 7, Krakow 30-387, Poland; andrzej.gorecki@uj.edu.pl
4 Department of Analysis and Differential Equations, Faculty of Mathematics and Computer Science, University of Warmia and Mazury, Słoneczna 54, PL-10-710 Olsztyn, Poland; aga.niemczynowicz@gmail.com
5 Department of Physical Chemistry–Interfacial Phenomena, Faculty of Chemistry, Maria Curie–Sklodowska University, 20-031 Lublin, Poland; aszczes@poczta.umcs.lublin.pl
6 Department of Applied Physics, Lublin University of Technology, Nadbystrzycka 38, 20-618 Lublin, Poland; g.gladyszewski@pollub.pl
7 Institute of Industrial Organic Chemistry, Annopol 6, 03-236 Warsaw, Poland; andrzej.niewiadomy@up.lublin.pl
8 Department of Chemistry, University of Life Sciences in Lublin, 20-950 Lublin, Poland
* Correspondence: arkadiusz.matwijczuk@up.lublin.pl; Tel.: +48-81-445-69-37

Received: 24 October 2018; Accepted: 31 October 2018; Published: date

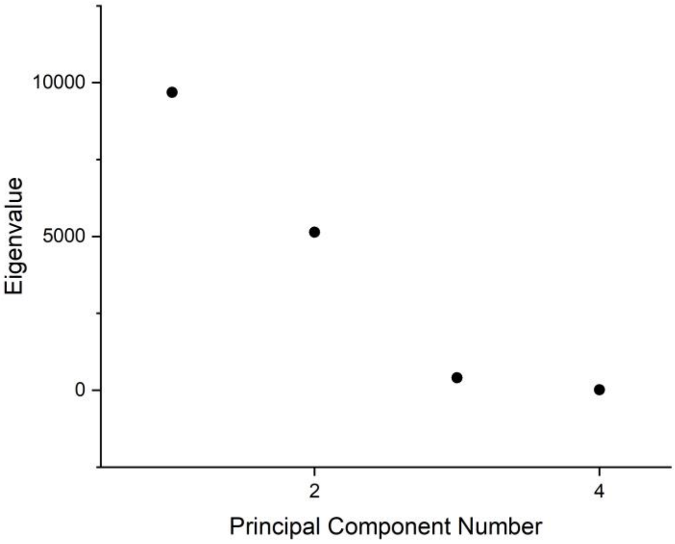

Figure S1. Contribution of eigenvalues with PCs.

(a)

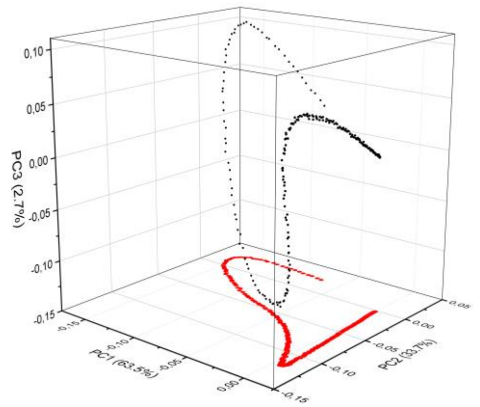

(b)

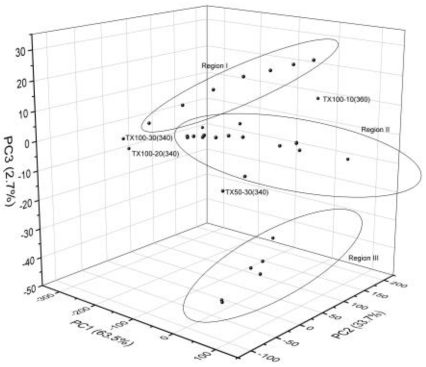

Figure S2. 3D plots (PC x PC2 x PC3) of loading (a) and score (b) from PCA.

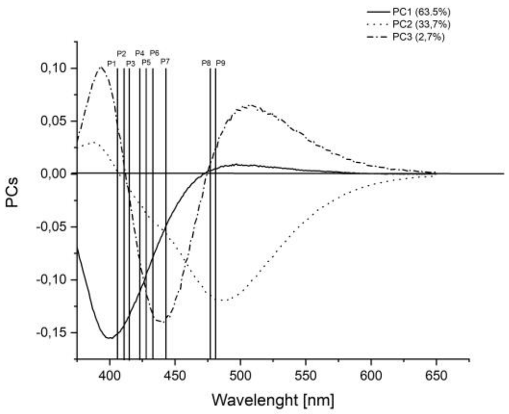

Figure S3. The loading vectors for PCs plotted as a function of wavelength. The plots indicate which spectral shape are associated with variance in the overall signal.

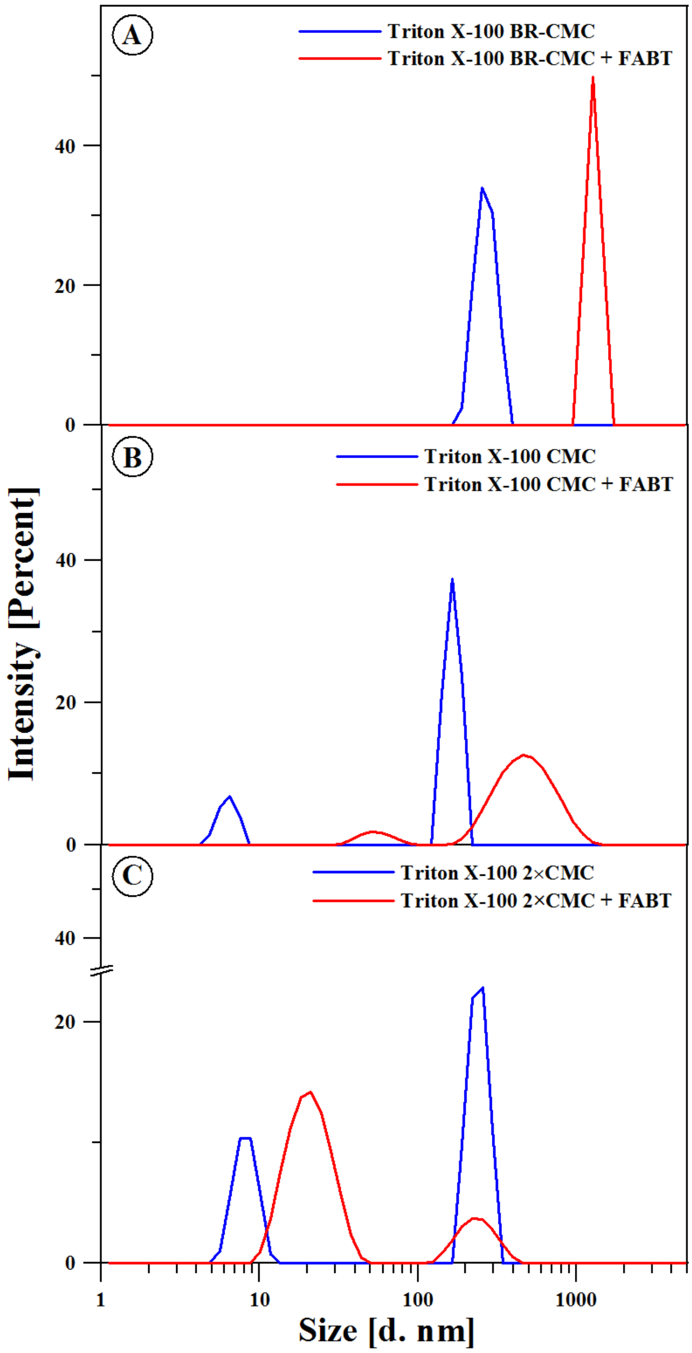

Figure S4. Distribution of DLS intensities measured in PBS buffer solutions of (a) Triton X-100 BR-CMC (blue line), Triton X-100 BR-CMC + FABT (red line) (b) Triton X-100 CMC (blue line), Triton X-100 CMC + FABT (red line) (c) Triton X-100 2×CMC (blue line), Triton X-100 2×CMC + FABT (red line).

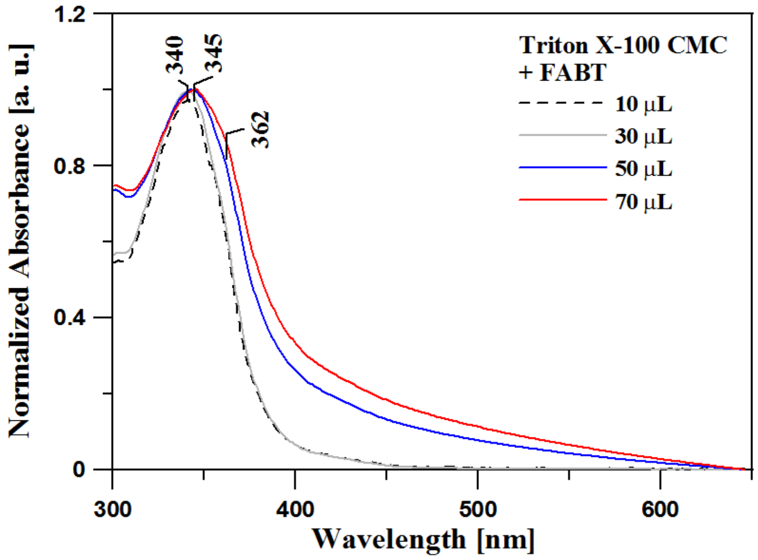

Figure S5. Normalized electronic absorption spectra for various amounts of FABT added in Mt-OH to the system with Triton X-100 detergent in the amount of 50μl per 3 ml of the buffer. The measurements were performed at the temperature of 23 °C.

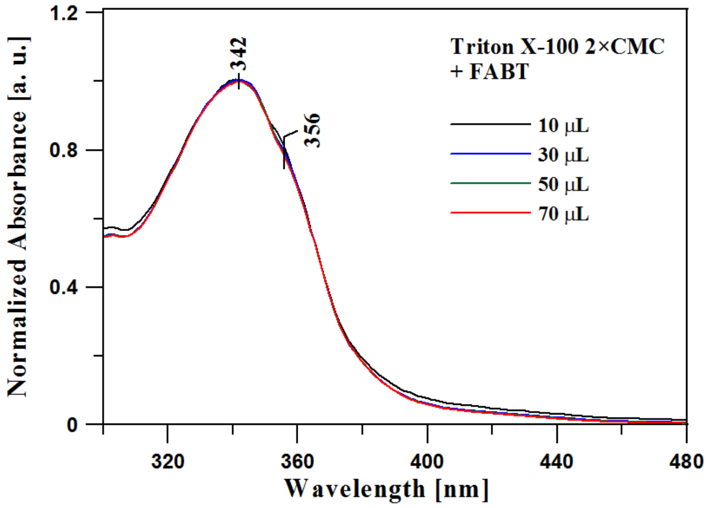

Figure S6. Normalized electronic absorption spectra for various amounts of FABT added in Mt-OH to the system with Triton X-100 detergent in the amount of 100μl per 3 ml of the buffer. The measurements were performed at the temperature of 23 °C.

Table S1. Results of Principal Component Analysis performed on dataset.

Principal Component Number	Eigenvalue	Percentage of Variance (%)	Cumulative (%)
1	9682, 43591	63, 4712	63, 4712
2	5141, 15864	33, 7018	97, 17301
3	406, 41907	2, 6642	99, 8372
4	15, 6595	0, 10265	99, 93985
